# Four new species of the subgenus *Minettiella* from China (Diptera, Lauxaniidae, *Minettia*)

**DOI:** 10.3897/zookeys.932.50763

**Published:** 2020-05-12

**Authors:** Wenliang Li, Xulong Chen, Ding Yang

**Affiliations:** 1 College of Forestry, Henan University of Science and Technology, Luoyang 471023, China Henan University of Science and Technology Luoyang China; 2 Department of Entomology, China Agricultural University, Beijing 100193, China China Agricultural University Beijing China

**Keywords:** *
Minettiella
*, new species, Oriental Region, southwest China

## Abstract

Four species of *Minettia* Robineau-Desvoidy, 1830 from Southwest China are described as new to science: Minettia (Minettiella) dashahensis**sp. nov.**, M. (Minettiella) longispina**sp. nov.**, M. (Minettiella) membranacea**sp. nov.** and M. (Minettiella) zhaotongensis**sp. nov.** A key to the species of the subgenus Minettiella of the world is presented.

## Introduction

The subgenus Minettiella Malloch, 1929 was erected for the type species *Lauxania atratula* Meijere, 1910 and another species *L. atrata* Meijere, 1924. Shatalkin (1993, 1996) described two new species Minettia (Minettiella) coracina and *Minettiella elbergi*. Sasakawa and Kozánek (1995) described two new species, *Calliopum acrostichalis* and *C. dolabriforme*, meanwhile, Sasakawa and Mitsui (1995) described a new species, *Minettiella japonica*. Shatalkin (2000) noted that *C. acrostichalis* Sasakawa & Mitsui, 1995 might be a synonym of Minettia (Minettiella) coracina Shatalkin, 1993, both *Minettiella japonica* Sasakawa & Mitsui, 1995 and *Minettiella elbergi* Shatalkin, 1996 might be synonyms of *Minettiella dolabriforme* (Sasakawa & Kozánek, 1995). Shi, Wang and Yang (2011) described two new species, Minettia (Minettiella) bawanglingensis and Minettia (Minettiella) clavata, from China and transferred Sapromyza (Sapromyza) acrostichalis Sasakawa, 2001 to this subgenus. Shi and Yang (2014) also described five new species from the South of China and presented a key to separate world species. To date, 15 species with four currently found in China are known throughout the world: 13 species are distributed in the Oriental Region, two species in the Palaearctic Region, and eleven species in China.

It is controversial whether Minettiella is a subgenus or a genus. Shatalkin (1996) raised the subgenus Minettiella to the genus level because of the specialized male genitalia and other diagnosis of *Minettiella elbergi* Shatalkin, 1996. Lee and Han (2014) consider that the genus *Minettiella* can only be distinguished from the closely resembling genera by the combination of the yellow body coloration and single katepisternal seta, and also pointed out that these characters do not seem to justify their generic differentiation. Shi and Yang (2014) examined the male genitalia of some specimens from five subgenera of *Minettia*, found the diversity of the male genitalia in *Minettiella* was the same as that in the subgenera *Minettia* and *Plesiominettia* of genus *Minettia*, and rejected Shatalkin’s elevation of the subgenus Minettiella to the genus level. We agree with Shi and Yang (2014) and insist that Minettiella is a subgenus of the genus Minettia.

The subgenus is recognized by anterior edge of frons glossy and upturned relative to rest of frons, face and parafacialia with gray pruinosity, shiny frons, hyaline yellow wings, scutum with intra-alar seta, katepisternum with a discal katepisternal seta, and mesonotum with 0–1 + 2–3 dorsocentral setae and 0–1 + 2–4 acrostichal setulae; most species have one to three pairs of strong acrostichal setulae (Malloch 1929; Stuckenberg 1971; Shatalkin 2000; Lee and Han 2014).

## Materials and methods

General terminology follows Cumming and Wood (2009) and Gaimari and Silva (2010). Genitalia preparations were made by removing and macerating the apical portion of the abdomen in cold saturated NaOH for six hours, then rinsing and neutralizing them for dissection and study. After examination in glycerin, they were transferred to fresh glycerin and stored in a microvial pinned below the specimen or moved to an ethanol tube together with the wet specimens. Specimens examined were deposited in the China Agricultural University, Beijing, China (**CAUC**).

## Taxonomy

### Key to the subgenera of *Minettia* and the species of the subgenus Minettiella

**Table:** 

1	Frons shining and face flat; mesonotum with 0–1 + 2–3 dorsocentral setae; katepisternum with one katepisternal seta; male genitalia: dorsal aedeagal sclerite present	subgenus Minettiella Malloch (5)
–	Frons often dull and face slightly concave; mesonotum with 0+3 dorsocentral setae; katepisternum with two katepisternal setae; male genitalia: dorsal aedeagal sclerite absent (if dorsal aedeagal sclerite present, but no presutural dorsocentral setae	2
2	Face with one pair of distinct oblong tumour processes on lower margin	3
–	Face flat, without tumour process on lower margin	4
3	Wing black at base; arista long plumose, the longest ray longer than wide of first flagellomere; postgonite with four asymmetric long or short sclerites	subgenus Frendelia Collin
–	Wing yellow at base; arista short plumose, the longest ray as long as half width of first flagellomere; postgonite with two asymmetric long or short sclerites	subgenus Scotominettia Shatalkin
4	Arista pubescent (some bare or short hair-like), wing yellow at base; postgonite consisting of one pair of sclerites, surrounding membranous aedeagus; aedeagal dorsal sclerite absent	subgenus Plesiominettia Shatalkin
–	Arista short plumose, wing dark yellow or pale brown at base; postgonite consisting of one pair of narrow and asymmetric sclerites in ventral view; aedeagal dorsal sclerite broad, quadrate, rectangular, triangular or trapeziform	subgenus Minettia Robineau-Desvoidy
5	Mesonotum with 1+3 dorsocentral setae, with long acrostichal setulae	6
–	Mesonotum lacking a presutural dorsocentral setae, without long acrostichal setulae	7
6	Arista bare; acrostichal setulae in two rows; male genitalia: anterior epandrium narrow and posterior broad, with a deep concavity on anterior ventral margin and a digitiform anterior process, triangular apically in lateral view; surstylus elliptical in lateral view	Minettia (Minettiella) atratula
–	Arista pubescent (Fig. 1); acrostichal setulae in four rows (Fig. 4); male genitalia (Figs 6–10): epandrium without deep concavity on anterior ventral margin and a digitiform anterior process in lateral view; surstylus not elliptical in lateral view	Minettia (Minettiella) dashahensis sp. nov.
7	Mesonotum with 0 + 2 dorsocentral setae	8
–	Mesonotum with 0 + 3 dorsocentral setae (exceptionally M. (Minettiella) dolabriforma rarely with 0 + 3 dorsocentral setae, anteriormost dorsocentral setae is considerably smaller than usual, only half length of the second dorsocentral setae	15
8	Mesonotum with acrostichal setulae in six rows (exceptionally M. (Minettiella) dolabriforma rarely with acrostichal setulae in six rows, a pair of acrostichal setulae long, just behind level of anterior dorsocentral setae and ca. 2/3 length of prescutellar seta)	9
–	Mesonotum with acrostichal setulae in four rows	13
9	Acrostichal setulae with two pairs of strong setae in front of a pair of prescutellar seta	Minettia (Minettiella) bawanglingensis
–	Acrostichal setulae with pair of strong setae in front of a pair of prescutellar setae	10
10	Arista plumose; only hind tarsomeres yellow	Minettia (Minettiella) atrata
–	Arista pubescent; legs not as above	11
11	Surstylus long, extended part as long as height of epandrium, expanded apically in lateral view (Fig. 36)	Minettia (Minettiella) zhaotongensis sp. nov.
–	Surstylus short, extended part short than half height of epandrium, slender apically in lateral view	12
12	Syntergosternite 7+8 with one anterior median process in lateral view (Fig. 28), one membranous band under ventral bridge; surstylus recurved in lateral view (Fig. 26); hypandrium V-shaped; postgonite curved outward apically in ventral view (Fig. 29), with long setulae; aedeagus with membranous processes internally (Fig. 30)	Minettia (Minettiella) membranacea sp. nov.
–	Syntergosternite 7+8 with one broad membranous process (Fig. 18); surstylus twisty in lateral view (Fig. 16); hypandrium narrow and U-shaped, with distinct membranous inner process (Fig. 19); aedeagus with long spines ventrally (Fig. 20)	Minettia (Minettiella) longispina sp. nov.
13	Arista with microscopic setulae, shorter than 1/4 height of first flagellomere; male genitalia: surstylus narrow apically with falcate apical process in lateral view	Minettia (Minettiella) sasakawai
–	Arista pubescent, with longest rays ca. 1/3 height of first flagellomere; male genitalia: surstylus wide apically with teeth or acute process in lateral view	14
14	Female sternite nine rectangular, ca. 3 × as wide as long, and sternite seven without triangular apical processes; male genitalia: surstylus with acute process projecting forwards in lateral view	Minettia (Minettiella) dolabriforma
–	Female sternite nine semicircular, sternite seven with pair of triangular apical processes; male genitalia: surstylus contorted claviform in lateral view and cone-shaped in posterior view, with two acute apical teeth	Minettia (Minettiella) tianmushanensis
15	Mesonotum with acrostichal setulae in two rows	16
–	Mesonotum with acrostichal setulae in six rows	17
16	Anepisternum with bluish grey pruinescence; mid and hind tibiae yellow	Minettia (Minettiella) acrostichalis
–	Anepisternum with whitish grey pruinescence; mid and hind tibiae yellow except blackish apical 1/4	Minettia (Minettiella) coracina
17	Mesonotum with brownish grey pruinescence, first postsutural dorsocentral setae weak, hairlike; male genitalia: surstylus fused with the epandrium, claviform with a triangular basal process, a projecting apical process, a small acute ventroapical process and a tiny incision in lateral view	Minettia (Minettiella) clavata
–	Mesonotum with whitish grey pruinescence, first postsutural dorsocentral setae strong; male genitalia: surstylus articulated with epandrium, triangular or lobe-like with a single process	18
18	Face and parafacial with sparse whitish gray pruinescence; arista short plumose, longest rays slightly shorter than height of first flagellomere; mid femur with four anterior setae; male genitalia: epandrium with wide median incision on posterior margin in lateral view; surstylus short cone-shaped and originating from inner side of epandrium in lateral view and converging apically in posterior view; female unknown	Minettia (Minettiella) plurifurcata
–	Face with a yellow triangular median spot or only slightly yellow at middle of face, and parafacial with dense whitish gray pruinescence; arista with microscopic setulae, longest rays shorter than 1/3 height of first flagellomere; mid femur with three anterior setae; male genitalia: epandrium with small subapical incision in lateral view and acute apically in posterior view, surstylus contorted with apical setulae and originating from inner side of epandrium in lateral view; female sternite eight semicircular with pair of processes on anterior margin and a wide groove between processes	Minettia (Minettiella) spinosa

### Species descriptions

#### Minettia (Minettiella) dashahensis sp. nov.

Taxon classificationAnimaliaDipteraLauxaniidae

FD2E2BE7-677D-5D69-B7AA-C0E7A061FE6C

http://zoobank.org/A7DB3137-7D60-41E8-BE8F-68807FE58095

Figures 1–5 , 6–10

##### Type material.

***Holotype***: ♂ (CAUC), China, Guizhou: Dashahe qianfengcun, 1300–1550 m, 18.viii.2004, Yajun Zhu. ***Paratypes***: 1♀(CAUC), China, Guizhou: as holotype; 1♂, 1♀(CAUC), China, Guizhou: Dashahe qianfengcun, 1300–1550 m, 19.viii.2004, Yajun Zhu; 1♀(CAUC), China, Guizhou: Dashahe qianfengcun, 1350–1500 m, 17.viii.2004, Yajun Zhu; 1♂, 1♀(CAUC), China, Guizhou: Dashahe hebacun, 1200–1500 m, 20.viii.2004, Yajun Zhu.

**Figures 1–5.**
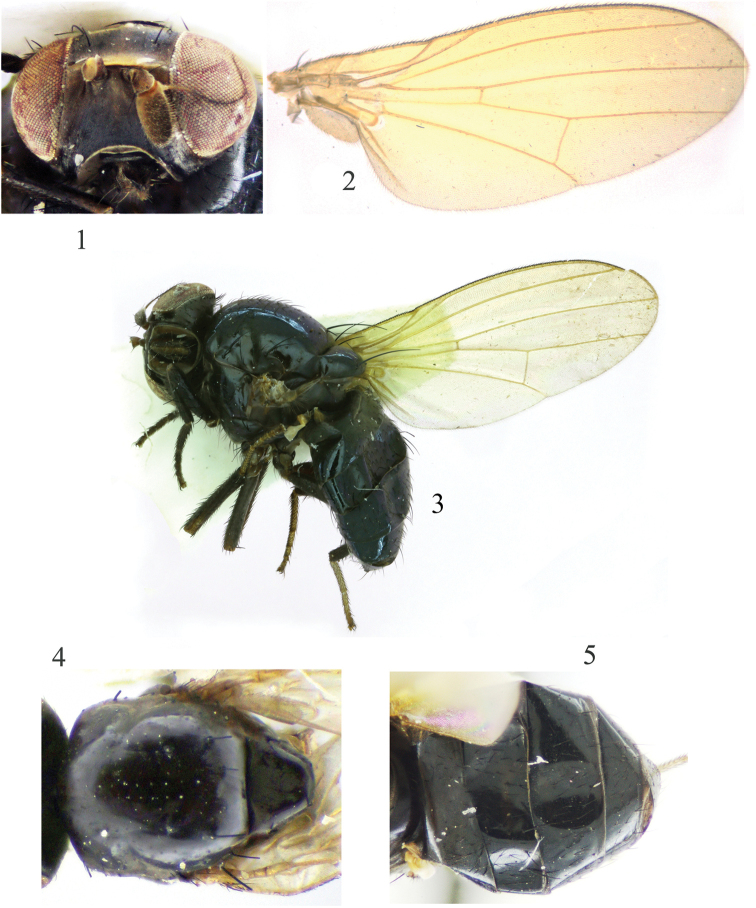
Minettia (Minettiella) dashahensis sp. nov. Male **1** head, anterior view **2** wing **3** habitus, lateral view **4** thorax, dorsal view **5** abdomen, dorsal view.

##### Etymology.

The specific epithet is named for holotype locality in Guizhou, Dashahe.

##### Diagnosis.

Arista blackish brown except paler basally. Mesonotum 1 + 3 dorsocentral setae, acrostichal setulae in four irregular rows, with two pairs of strong acrostichal setulae before prescutellar setae. Epandrium broad, ventral apical angle with setae. Surstylus separated from epandrium and extended from interior of epandrium, curved in lateral view, with connective sclerite under cercus in posterior view. Aedeagal apodeme long, nearly as long as aedeagus.

##### Description.

**Male.** Body length 3.4–3.5 mm, wing length 3.7–3.8 mm. **Female.** Body length 3.0–3.4 mm, wing length 3.5–3.8 mm.

***Head*** (Fig. 1) black. Face and parafacial with dense grayish white pruinescence. Frons blackish brown, anterior margin yellow; ocellar triangle black, ocellar setae developed, almost as long as posterior fronto-orbital setae and extending to anterior margin of frons; anterior fronto-orbital setae reclinate, shorter than posterior fronto-orbital setae. Gena ca. 1/6 height of eye. ***Antenna*** brownish yellow, first flagellomere blackish brown on apical 2/3 of dorsal margin, 1.7 times longer than high; arista blackish brown except paler basally, plumose with longest setula slightly longer than height of first flagellomere. Proboscis blackish brown, with pale yellow and black setulae; palpus black with black setulae.

***Thorax*** (Fig. 4) black, with gray pruinescence. 1 + 3 dorsocentral setae, acrostichal setulae in four irregular rows, with two pairs of strong acrostichal setulae before prescutellar setae; a pair of prescutellar setae, shorter than anteriormost dorsocentral setae. One anepisternal seta and one katepisternal seta. ***Legs*** black, tibia brownish yellow at base, mid and hind tarsomeres dark yellow (sometimes fore tarsomeres also dark yellow). Fore femur with eight posterior dorsal setae and five posterior ventral setae; fore tibia with one dorsal preapical seta and one short apical ventral seta. Mid femur with three or four anterior setae and one short apical posterior seta; mid tibia with one strong dorsal preapical seta and one strong apical ventral seta. Hind femur with one preapical anterior dorsal seta; hind tibia with one weak dorsal preapical seta and one short apical ventral seta. ***Wing*** slight yellow and hyaline, pale yellow at base; costa with 2^nd^ (between R_1_ and R_2+3_), 3^rd^ (between R_2+3_ and R_4+5_), and 4^th^ (between R_4+5_ and M_1_) sections in proportion of 9.5 : 2.8 : 2.1; r-m on middle of discal cell; ultimate and penultimate sections of M_1_ in proportion of 6.8 : 3.9; ultimate section of CuA_1_ ca. 1/5 of penultimate. Haltere pale yellow.

**Figures 6–10.**
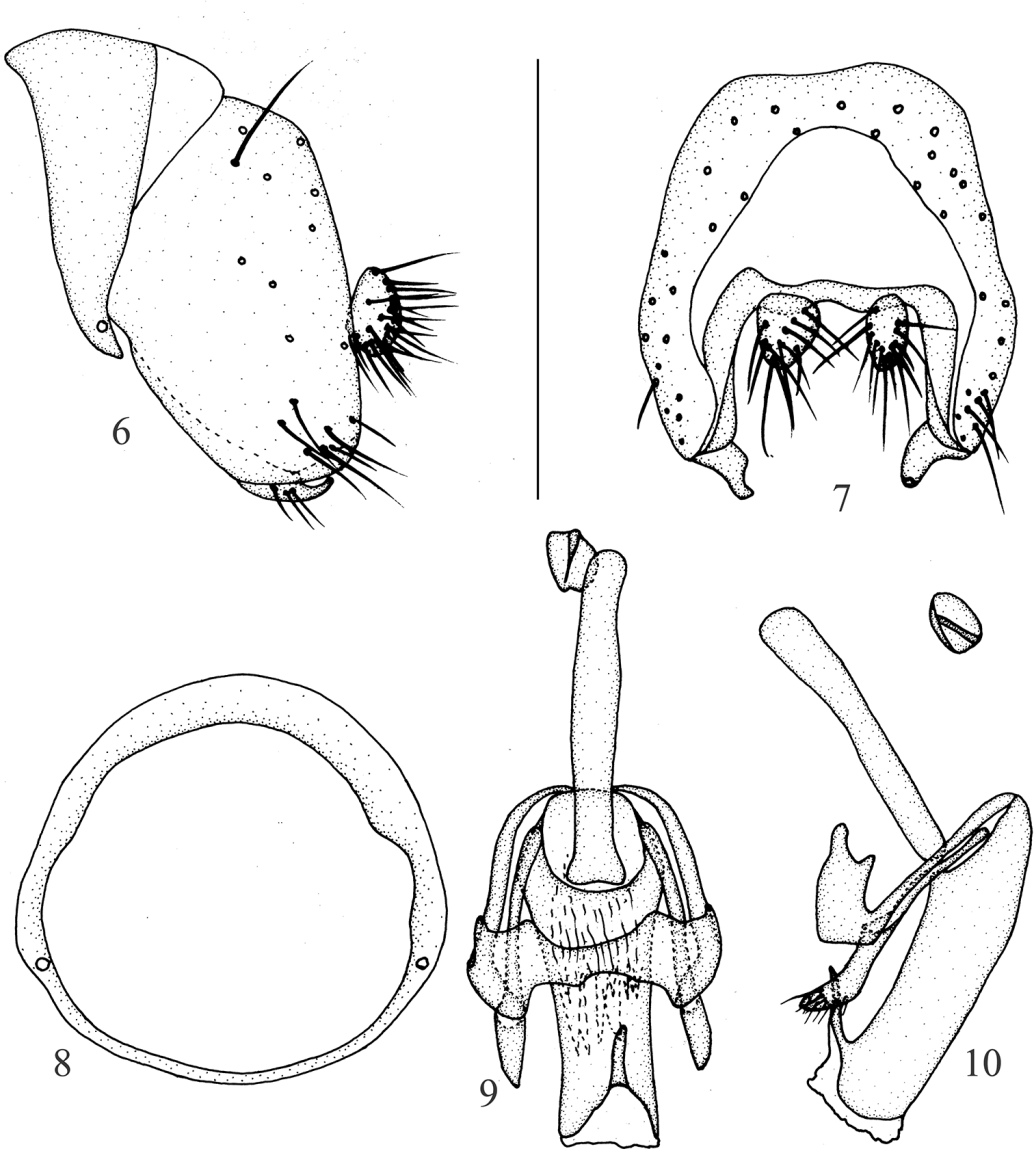
Minettia (Minettiella) dashahensis sp. nov. Male **6** syntergosternite and epandrium, lateral view **7** epandrial complex, posterior view **8** syntergosternite, anterior view **9** aedeagal complex, ventral view **10** aedeagal complex, lateral view. Scale bar: 0.5 mm.

***Abdomen*** (Fig. 5) black, with sparse gray pruinescence. ***Male genitalia*** (Figs 6–10): syntergosternite circular. Epandrium broad, ventral apical angle with setae. Surstylus separated from epandrium and extended from interior of epandrium, tapering apically, curved in lateral view, with connective sclerite under cercus in posterior view; hypandrium narrow in the middle and wide on both sides, with small inner process. Postgonite with setae, slender and curved apically in lateral view; aedeagus tubular, ventral sclerite V-shaped concave apically in ventral view, with a single long spiny process on the bottom, forming an acute angle with aedeagus, aedeagus with membranous processes internally; aedeagal apodeme as long as aedeagus.

##### Remarks.

The new species is very similar to M. (Minettiella) sasakawai from China (Hainan) and Vietnam in the following characteristics: acrostichal setulae in four rows; mid and hind tarsomeres dark yellow, fore femur with eight posterior dorsal setae and five posterior ventral setae; wing slight yellow and hyaline, pale yellow at base; hypandrium narrow in the middle and wide on both sides, but it can be separated from the latter in the mesonotum with 1+3 dorsocentral setae, two pairs of strong acrostichal setulae present in front of prescutellar setae; the syntergosternite being circular; epandrium is broad. In M. (Minettiella) sasakawai, the mesonotum with 0 + 2 dorsocentral setae, a pair of strong acrostichal setulae present in front of prescutellar setae; the syntergosternite is semicircular and narrow under the spiracle; epandrium is slender.

##### Distribution.

China (Guizhou).

#### Minettia (Minettiella) longispina sp. nov.

Taxon classificationAnimaliaDipteraLauxaniidae

5DEB2B5C-5A5E-52BA-BC9B-ADAE2D8E4500

http://zoobank.org/1B69B24A-2064-4546-8EDC-455245FBE1E8

Figures 11–15 , 16–20

##### Type material.

***Holotype***: ♂ (CAUC), China, Guizhou: Fanjing mountain, 1300–1900 m, 29.vii.2001, Fang Zhao. ***Paratypes***: 1♂, 5♀♀ (CAUC), China, Guizhou: Fanjing mountain, 1750–2200 m, 1.viii.2001, Fang Zhao; 1♂ (CAUC), China, Guizhou: Fanjing mountain, 950–1750 m, 2.viii.2001, Fang Zhao; 4♀♀ (CAUC), China, Guizhou: Fanjing mountain, 1300–1900 m, 29.vii.2001, Caixia Gao; 1♀ (CAUC), China, Guizhou: Fanjing mountain, 1900–2490 m, 30.vii.2001, Caixia Gao; 3♀♀ (CAUC), China, Guizhou: Fanjing mountain, 1750–2000 m, 1.viii.2001, Caixia Gao; 1♂6♀♀(CAUC), China, Guizhou: Fanjing mountain, 950–1750 m, 2.viii.2001, Caixia Gao; 1♀ (CAUC), China, Guizhou: Fanjing mountain, 1300 m, 27.vii.2001, Wanzhi Cai.

**Figures 11–15.**
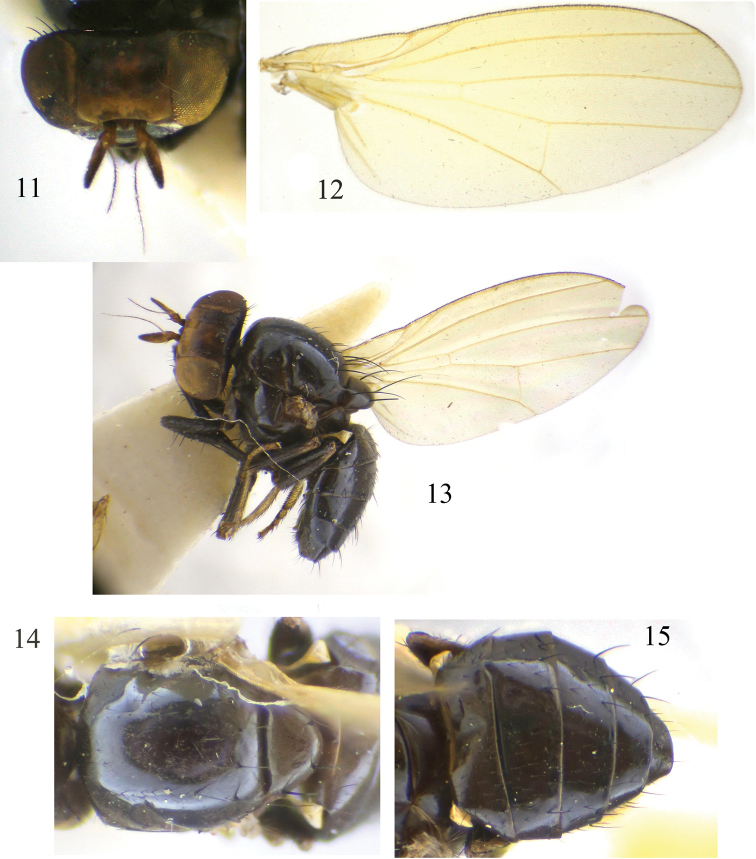
Minettia (Minettiella) longispina sp. nov. Male **11** head, anterior view **12** wing **13** habitus, lateral view **14** thorax, dorsal view **15** abdomen, dorsal view.

##### Etymology.

Latin, *longispina*, referring to the aedeagus with a long membranous spin-like processes internally.

##### Diagnosis.

Frons dark brown, anterior margin yellow, two black longitudinal stripes along fronto-orbital rows, extending from ocellar triangle to occiput. Arista blackish brown except paler basally, pubescent with longest setulae almost as long as 1/4 height of first flagellomere. Mesonotum with 0 + 2 dorsocentral setae, acrostichal setulae in six irregular rows. Surstylus separated from epandrium, distorted in lateral view and only one short curved claviform process, narrow apically and inwards curved in posterior view. Aedeagal apodeme long, nearly as long as aedeagus.

##### Description.

**Male.** Body length 3.6–3.8 mm, wing length 4.0–4.2 mm. **Female.** Body length 3.8–4.0 mm, wing length 4.0–4.5 mm.

***Head*** (Fig. 11) black. Face and parafacial with dense grayish white pruinescence. Frons dark brown, anterior margin yellow, as long as wide and parallel-sided, two black longitudinal stripes along fronto-orbital rows, extending from ocellar triangle to occiput; ocellar triangle black, ocellar setae developed, almost as long as posterior fronto-orbital setae and extending to anterior margin of frons; anterior fronto-orbital setae reclinate, shorter than posterior fronto-orbital setae. Gena ca. 1/6 height of eye. ***Antenna*** brownish yellow, first flagellomere blackish brown on 2/3 apex of dorsal margin, 1.7 times longer than high; arista blackish brown except paler basally, pubescent with longest setulae almost as long as 1/4 height of first flagellomere. Proboscis blackish brown, with pale yellow and black setulae; palpus black with black setulae.

***Thorax*** (Fig. 14) black, with gray pruinescence. 0 + 2 dorsocentral setae, acrostichal setulae in six irregular rows, with pair of strong acrostichal setulae before prescutellar setae; a pair of prescutellar setae, almost as long as anteriormost dorsocentral setae. One anepisternal seta and one katepisternal seta. ***Legs*** black, tibia brownish yellow at base, mid and hind tarsomeres dark yellow (sometimes fore tarsomeres also dark yellow). Fore femur with eight posterior dorsal setae and five posterior ventral setae; fore tibia with one dorsal preapical seta and one short apical ventral seta. Mid femur with four anterior setae and one short apical posterior seta; mid tibia with one strong dorsal preapical seta and one strong apical ventral seta. Hind femur with one preapical anterior dorsal seta; hind tibia with one weak dorsal preapical seta and one short apical ventral seta. ***Wing*** slight yellow and hyaline, pale yellow at base; costa with 2^nd^ (between R_1_ and R_2+3_), 3^rd^ (between R_2+3_ and R_4+5_), and 4^th^ (between R_4+5_ and M_1_) sections in proportion of 7.1 : 2.0 : 1.5; r-m on middle of discal cell; ultimate and penultimate sections of M_1_ in proportion of 4.9 : 2.8; ultimate section of CuA_1_ ca. 1/6 of penultimate. Haltere pale yellow.

**Figures 16–20.**
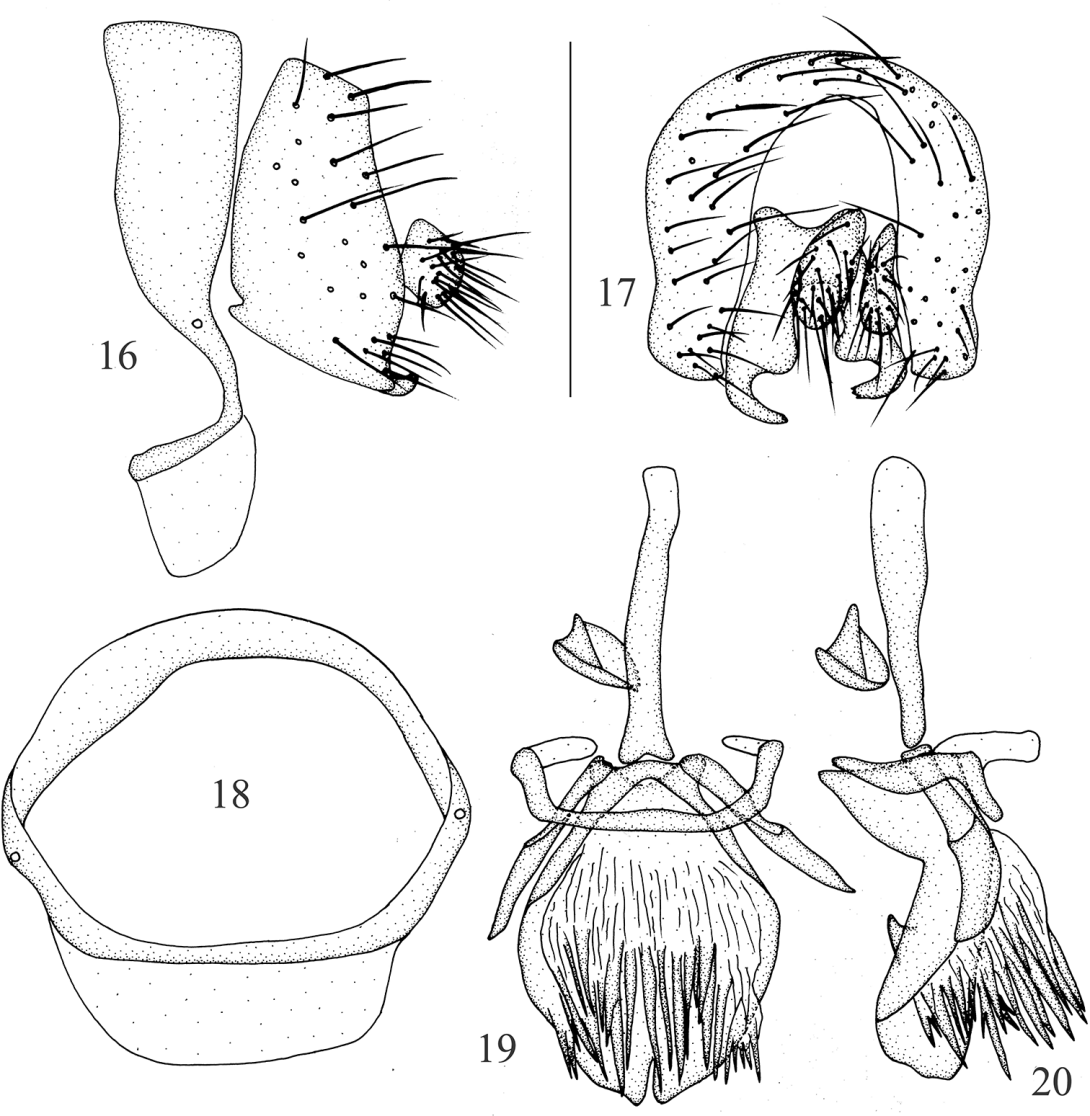
Minettia (Minettiella) longispina sp. nov. Male **16** syntergosternite and epandrium, lateral view **17** epandrial complex, posterior view **18** syntergosternite, anterior view **19** aedeagal complex, ventral view **20** aedeagal complex, lateral view. Scale bar: 0.5 mm.

***Abdomen*** (Figs 15) black, with sparse gray pruinescence. ***Male genitalia*** (Figs 16–20): syntergosternite circular, with one broad membranous ventral process. Epandrium narrow dorsally and broad ventrally, with one small ventral concave basally, ventral setae apically. Surstylus separated from epandrium, distorted in lateral view and only saw one short curved claviform process, narrow apically and inwards curved in posterior view, with connective broad sclerite under cercus at base; hypandrium narrow, U-shaped, with one distinct membranous inner process. Postgonite slender in ventral view, acute at tip, as long as half-length of aedeagus; aedeagus with long spiny processes internally, with distinct apical concave; aedeagal apodeme long, nearly as long as aedeagus.

##### Remarks.

The new species is very similar to Minettia (Minettiella) membranacea sp. nov. from Yunnan in the following characteristics: frons with two black longitudinal stripes along fronto-orbital rows, extending from ocellar triangle to occiput; mesonotum with 0 + 2 dorsocentral setae, acrostichal setulae in six irregular rows, and a pair of strong acrostichal setulae present in front of prescutellar setae; wing slight yellow and hyaline, pale yellow at base, but it can be separated from the latter in the fore femur with five posterior ventral setae, the mid femur with four anterior setae, the syntergosternite with one broad membranous process; the hypandrium narrow and U-shaped; the aedeagus with long spines ventrally. In M. (Minettiella) membranacea sp. nov., the fore femur with six posterior ventral setae, the mid femur with three anterior setae, the syntergosternite with one anterior median process in lateral view, one membranous band under ventral bridge; the hypandrium V-shaped; the aedeagus with membranous processes internally.

##### Distribution.

China (Guizhou).

#### Minettia (Minettiella) membranacea sp. nov.

Taxon classificationAnimaliaDipteraLauxaniidae

60AD4A4C-4806-5087-A791-B7B574878900

http://zoobank.org/418711C9-1429-4368-8E48-5BC58D852241

Figures 21–25 , 26–30

##### Type material.

***Holotype***: ♂ (CAUC), China, Yunnan: Baoshan Baihualing hot spring, 1500 m, 29.v.2007, Xingyue Liu.

##### Etymology.

Latin, *membranacea*, referring to the aedeagus with membranous processes internally.

**Figures 21–25.**
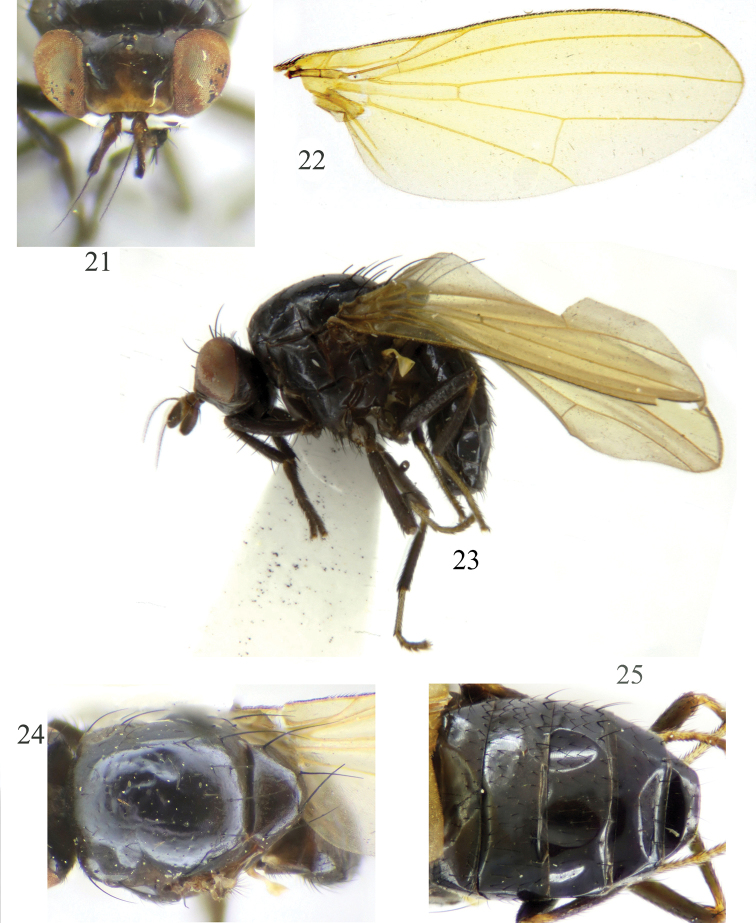
Minettia (Minettiella) membranacea sp. nov. Male **21** head, anterior view **22** wing **23** habitus, lateral view **24** thorax, dorsal view **25** abdomen, dorsal view.

##### Diagnosis.

Antenna brownish yellow, first flagellomere blackish brown on 2/3 apex of dorsal margin. Proboscis blackish brown, with pale yellow and black setulae. Mesonotum with two dorsocentral setae, acrostichal setulae in six irregular rows. Mid and hind tibia brownish yellow at base and tarsomeres dark yellow. Syntergosternite circular, broad dorsally and narrow ventrally, with one small anterior median process in lateral view, one membranous band under ventral bridge. Hypandrium median ventral side outward, V-shaped. Aedeagal apodeme long, slightly shorter than aedeagus.

##### Description.

**Male.** Body length 3.2 mm, wing length 4.3 mm. **Female.** Unknown.

***Head*** (Fig. 21) black. Face and parafacial with dense grayish white pruinescence. Frons dark brown, as long as wide and parallel-sided, two wide black longitudinal stripes along fronto-orbital rows, extending from ocellar triangle to occiput; ocellar triangle black, ocellar setae developed, almost as long as posterior fronto-orbital setae and extending to anterior margin of frons; anterior fronto-orbital setae reclinate, shorter than posterior fronto-orbital setae. Gena ca. 1/7 height of eye. ***Antenna*** brownish yellow, first flagellomere blackish brown on 2/3 of the apex of dorsal margin, 1.7 times longer than high; arista blackish brown except paler basally, pubescent with longest setula almost as long as 1/3 height of first flagellomere. Proboscis blackish brown, with pale yellow and black setulae; palpus black with black setulae.

***Thorax*** (Fig. 24) black, with brownish gray pruinescence. Two dorsocentral setae, acrostichal setulae in six irregular rows, with pair of strong acrostichal setulae before prescutellar setae; a pair of prescutellar setae, longer than anteriormost dorsocentral setae. One anepisternal seta and one katepisternal seta. ***Legs*** black, mid and hind tibia brownish yellow at base and tarsomeres dark yellow. Fore femur with eight posterior dorsal setae and six posterior ventral setae; fore tibia with one dorsal preapical seta and one short apical ventral seta. Mid femur with three anterior setae and one short apical posterior seta; mid tibia with one strong dorsal preapical seta and one strong apical ventral seta. Hind femur with one preapical anterior dorsal seta; hind tibia with one weak dorsal preapical seta and one short apical ventral seta. ***Wing*** slight yellow and hyaline, pale brown at base; costa with 2^nd^ (between R_1_ and R_2+3_), 3^rd^ (between R_2+3_ and R_4+5_), and 4^th^ (between R_4+5_ and M_1_) sections in proportion of 9.6 : 2.4 : 1.8; r-m before middle of discal cell; ultimate and penultimate sections of M_1_ in proportion of 6.4 : 4.4; ultimate section of CuA_1_ ca. 1/6 of penultimate. Haltere pale yellow.

**Figures 26–30.**
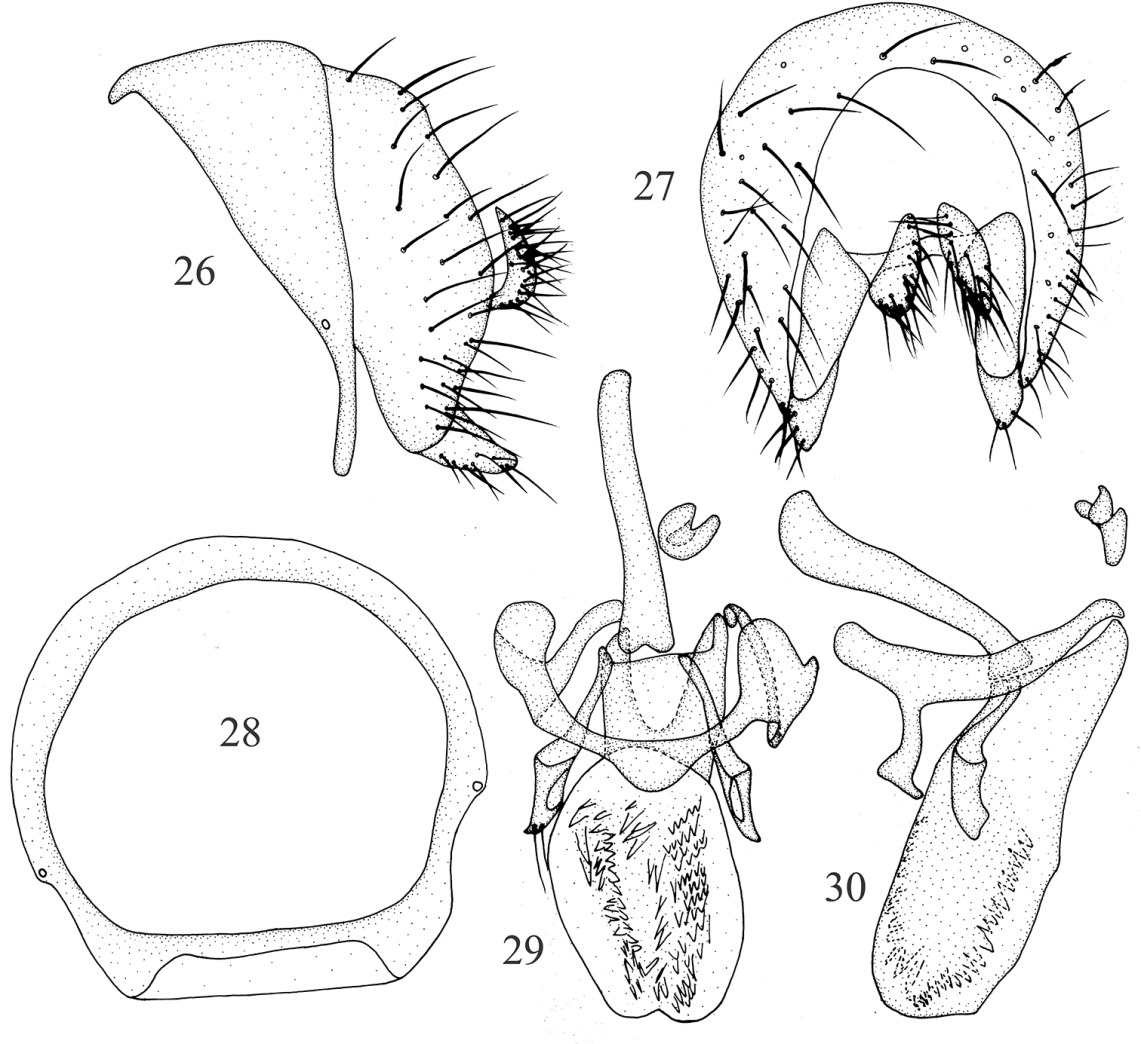
Minettia (Minettiella) membranacea sp. nov. Male **26** syntergosternite and epandrium, lateral view **27** epandrial complex, posterior view **28** syntergosternite, anterior view **29** aedeagal complex, ventral view **30** aedeagal complex, lateral view. Scale bar: 0.5 mm.

***Abdomen*** (Fig. 25) black, with sparse gray pruinescence. ***Male genitalia*** (Figs 26–30): syntergosternite circular, broad dorsally and narrow ventrally, with one small anterior median process in lateral view, one membranous band under ventral bridge. Epandrium slender. Surstylus separated from epandrium, with setae and curved in lateral view, with connective sclerite under cercus in posterior view; hypandrium median ventral side outward, V-shaped. Postgonite curved apically in ventral view, with long setae and nearly digitiform, aedeagus with membranous processes internally, with small apical concave; aedeagal apodeme long, slightly shorter than aedeagus.

##### Remarks.

The new species is very similar to Minettia (Minettiella) spinosa Shi & Yang from Hubei in the following characteristics: acrostichal setulae in six rows; the tarsomeres dark yellow, mid femur with three anterior setae and one short apical posterior seta; wing slight yellow and hyaline, but it can be separated from the latter in the face without spot; the mesonotum with pair of strong acrostichal setulae; the syntergosternite without setula near spiracle; the epandrium without subapical concavity; the hypandrium V-shaped. In M. (Minettiella) spinosa, the face with a yellow triangular median spot or only slightly yellow in center of face; the mesonotum without strong acrostichal setulae; the syntergosternite with one setula near spiracle; the epandrium with one small subapical concavity; the hypandrium H-shaped.

##### Distribution.

China (Yunnan).

#### Minettia (Minettiella) zhaotongensis sp. nov.

Taxon classificationAnimaliaDipteraLauxaniidae

B87934DF-3E66-559E-92BE-8E231D994AB0

http://zoobank.org/882CF48D-6D69-44E1-8C48-9CF08948260B

Figures 31–35 , 36–40

##### Type material.

***Holotype***: ♂(CAUC), China, Yunnan: Zhaotong Xiaocaoba, 1715 m, 15.ix.2009, Tingting Zhang. ***Paratypes***: 1♀ (CAUC), China, Yunnan: as holotype; 1♂, 1♀ (CAUC), China, Yunnan: Zhaotong Xiaocaoba, 1715 m, 15.ix.2009, weina Cui; 1♀ (CAUC), China, Yunnan: Zhaotong Xiaocaoba, 1900 m, 15.ix.2009, weina Cui; 1♂, 1♀ (CAUC), China, Yunnan: Kunming Heilongtan, 2016 m, 23.vii.2006, Kuiyan Zhang; 1♀ (CAUC), China, Yunnan: Kunming Heilongtan, 2016 m, 23.vii.2006, Wenliang Li.

##### Etymology.

The specific epithet is named for the holotype locality in Yunnan, Zhaotong.

##### Diagnosis.

Face and parafacial with dense grayish white pruinescence. Anterior fronto-orbital setae reclinate, shorter than posterior fronto-orbital setae. Mesonotum with 0+2 dorsocentral setae, acrostichal setulae in six irregular rows. Mid and hind tarsomeres dark yellow (sometimes fore tarsomeres also dark yellow). Wing slight yellow and hyaline, brown at base. Dorsal of epandrium narrower than dorsal of syntergosternite. Surstylus separated from epandrium, claviform, with single long seta in the middle, expanded apicially in lateral view and cone-shaped in posterior view. Postgonite hairy. Aedeagal apodeme tubular, as long as half-length of aedeagus.

**Figures 31–35.**
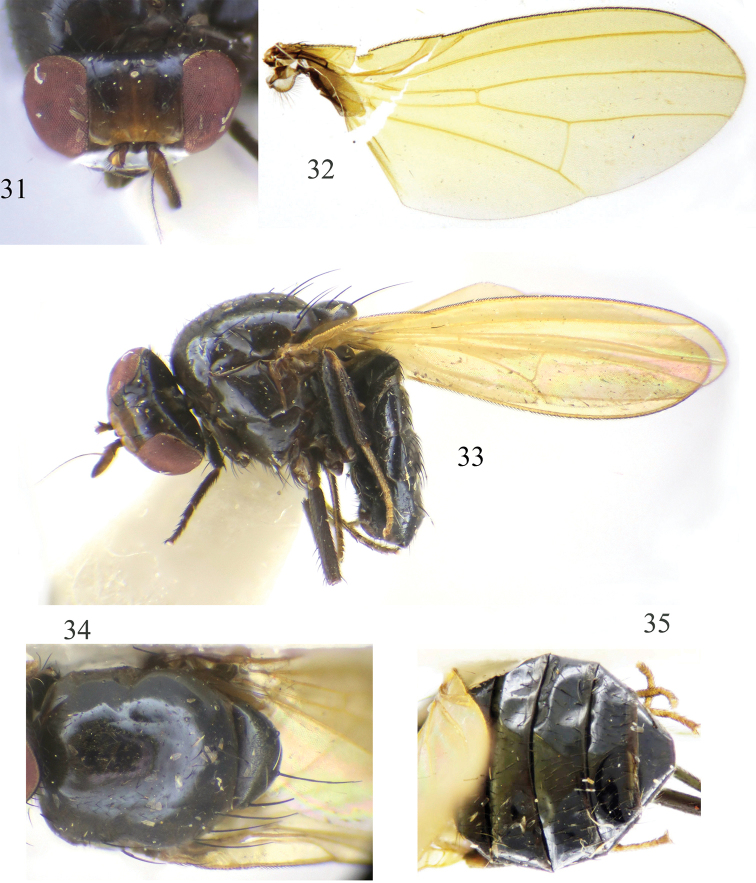
Minettia (Minettiella) zhaotongensis sp. nov. Male **31** head, anterior view **32** wing **33** habitus, lateral view **34** thorax, dorsal view **35** abdomen, dorsal view.

##### Description.

**Male.** Body length 3.9–4.2 mm, wing length 4.4–4.8 mm. **Female.** Body length 3.8–4.3 mm, wing length 4.3–4.9 mm.

***Head*** (Fig. 31) black. Face and parafacial with dense grayish white pruinescence. Frons dark brown, as long as wide and parallel-sided, two black longitudinal stripes along fronto-orbital rows, extending from ocellar triangle to occiput; ocellar triangle black, ocellar setae developed, longer than anterior fronto-orbital setae and posterior fronto-orbital setae, extending to anterior margin of frons; anterior fronto-orbital setae reclinate, shorter than posterior fronto-orbital setae. Gena ca. 1/6 height of eye. ***Antenna*** brownish yellow, first flagellomere blackish brown on 2/3 apex of dorsal margin, 1.7 times longer than high; arista blackish brown except paler basally, pubescent with longest setulae almost as long as 1/4 height of first flagellomere. Proboscis blackish brown, with pale yellow and black setulae; palpus black with black setulae.

***Thorax*** (Fig. 34) black, with gray pruinescence. 0+2 dorsocentral setae, acrostichal setulae in six irregular rows, with pair of strong acrostichal setulae before prescutellar setae; a pair of prescutellar setae, almost as long as anteriormost dorsocentral setae. One anepisternal seta and one katepisternal seta. ***Legs*** black, tibia brownish yellow at base (sometimes tibia dark yellow), mid and hind tarsomeres dark yellow (sometimes fore tarsomeres also dark yellow). Fore femur with eight posterior dorsal setae and five posterior ventral setae; fore tibia with one dorsal preapical seta and one short apical ventral seta. Mid femur with three or four anterior setae and one short apical posterior seta; mid tibia with one strong dorsal preapical seta and one strong apical ventral seta. Hind femur with one preapical anterior dorsal seta; hind tibia with one weak dorsal preapical seta and one short apical ventral seta. ***Wing*** slight yellow and hyaline, brown at base; costa with 2^nd^ (between R_1_ and R_2+3_), 3^rd^ (between R_2+3_ and R_4+5_), and 4^th^ (between R_4+5_ and M_1_) sections in proportion of 9.6 : 2.5 : 1.7; r-m beyond middle of discal cell; ultimate and penultimate sections of M_1_ in proportion of 6.5 : 3.7; ultimate section of CuA_1_ ca. 1/5 of penultimate. Haltere pale yellow.

**Figures 36–40.**
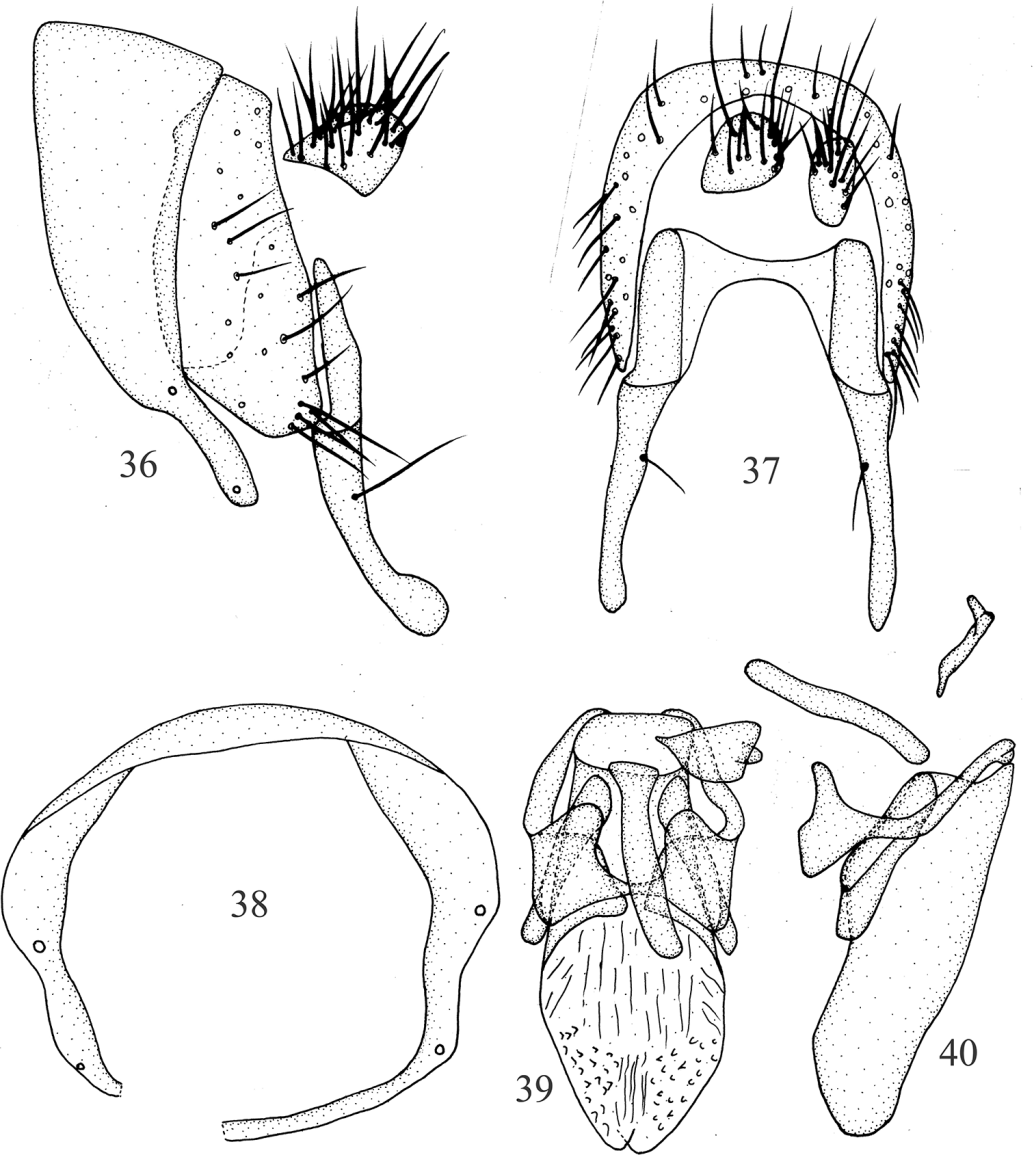
Minettia (Minettiella) zhaotongensis sp. nov. Male **36** syntergosternite and epandrium, lateral view **37** epandrial complex, posterior view **38** syntergosternite, anterior view **39** aedeagal complex, ventral view **40** aedeagal complex, lateral view. Scale bar: 0.5 mm.

***Abdomen*** (Fig. 35) black, with sparse gray pruinescence. ***Male genitalia*** (Figs 36–40): syntergosternite circular, broad dorsally and narrow ventrally. Dorsal of epandrium narrower than dorsal of syntergosternite. Surstylus separated from epandrium, claviform, with a long seta in the middle, expanded apically in lateral view and cone-shaped in posterior view, with connective weakly sclerotized claviform sclerite; hypandrium wide on both sides and concave in the middle, U-shaped. Postgonite hairy. Aedeagus with pair of ventral sclerites on basal 1/3 in ventral view, with membranous spiny processes apically, with small apical concave; aedeagal apodeme tubular, as long as half length of aedeagus.

##### Remarks.

The new species is very similar to Minettia (Minettiella) tianmushanensis Shi & Yang from Hainan in the following characteristics: mesonotum with 0 + 2 dorsocentral setae, a pair of strong acrostichal setulae present in front of prescutellar setae; the mid femur with three or four anterior setae; the surstylus is claviform, cone-shaped in posterior view, but it can be separated from the latter in the frons with two black longitudinal stripes along fronto-orbital rows; acrostichal setulae in six irregular rows; aedeagal apodeme as long as half length of aedeagus. In M. (Minettiella) tianmushanensis, the frons without black longitudinal stripe; acrostichal setulae in four irregular rows; aedeagal apodeme as long as aedeagus.

The new species is very similar to Minettia (Minettiella) membranacea sp. nov. from Yunnan in the following characteristics: frons with two black longitudinal stripes along fronto-orbital rows, extending from ocellar triangle to occiput; mesonotum with 0 + 2 dorsocentral setae, acrostichal setulae in six irregular rows, and a pair of strong acrostichal setulae present in front of prescutellar setae; wing slight yellow and hyaline, pale yellow at base, but it can be separated from the latter in the fore femur with five posterior ventral setae, the mid femur with four anterior setae, the syntergosternite with one broad membranous process; the hypandrium narrow and U-shaped; the aedeagus with long spines ventrally. In M. (Minettiella) membranacea sp. nov., the fore femur with six posterior ventral setae, the mid femur with three anterior setae, the syntergosternite with one anterior median process in lateral view, one membranous band under ventral bridge; the hypandrium V-shaped; the aedeagus with membranous processes internally.

##### Distribution.

China (Yunnan).

## Supplementary Material

XML Treatment for Minettia (Minettiella) dashahensis

XML Treatment for Minettia (Minettiella) longispina

XML Treatment for Minettia (Minettiella) membranacea

XML Treatment for Minettia (Minettiella) zhaotongensis
